# Optimizing *in vitro* embryo production in cattle: strategies for donor management and selection

**DOI:** 10.1590/1984-3143-AR2025-0079

**Published:** 2025-08-19

**Authors:** Alvaro García-Guerra, Jessica Cristina Lemos Motta, Rodrigo Vasconcellos Sala, Cameron Brontz Hayden, Eduardo Ponte, Victor Antonio Absalon-Medina, Pablo Juan Ross

**Affiliations:** 1 Department of Animal Sciences, The Ohio State University, Columbus, OH, USA; 2 School of Food and Agriculture, The University of Maine, Orono, ME, USA; 3 STgenetics, The Ohio Heifer Center, South Charleston, OH, USA; 4 STgenetics, Headquarters, Navasota, TX, USA

**Keywords:** *in vitro* embryo production, embryo transfer, FSH, ovarian superstimulation, bovine

## Abstract

*In vitro* embryo production (IVEP) has become a cornerstone of genetic advancement in cattle, yet its efficiency remains suboptimal and highly variable. This review synthesizes current knowledge on donor selection and management strategies aimed at optimizing IVEP outcomes. Central to IVEP success is the quantity and developmental competence of oocytes, which is influenced by both intrinsic donor characteristics and extrinsic management interventions. Ovarian superstimulation using follicle-stimulating hormone (FSH) has emerged as a key strategy to enhance oocyte yield and quality, with evidence supporting dose-dependent improvements in embryo development and yield. Protocol refinements—including timing, duration, and delivery mode of FSH— can further influence IVEP efficacy. Donor-specific factors such as age, pregnancy status, and size of the ovarian reserve, assessed via antral follicle count (AFC) or anti-Müllerian hormone (AMH) concentrations, significantly affect oocyte competence and/or embryo yield. Additionally, newly developed genomic traits and selection indexes, offer predictive value for donor performance and enable integration of IVEP-specific traits into breeding programs. High AMH donors consistently outperform low AMH counterparts, and emerging evidence suggests that tailoring superstimulation protocols to AMH phenotype can further enhance IVEP outcomes. The integration of physiological and genomic data provides the opportunity for developing targeted, phenotype/genotype-driven superstimulation protocols to maximize IVEP efficiency in a cost-effective and biologically sound manner.

## Introduction

The embryo transfer (ET) industry has evolved remarkably since Walter Heape successfully transferred embryos in rabbits (Heape and Foster, 1891), from a largely experimental technique to an essential tool for genetic advancement in cattle. The success of human *in vitro* fertilization (IVF) – highlighted by the birth of Louise Brown in 1978, the first human born through IVF (Steptoe and Edwards, 1978) – fueled efforts to develop *in vitro* embryo production (IVEP) systems in livestock. Although the birth of the first IVF calf in 1982 highlighted the potential of IVEP in cattle (Brackett et al., 1982), methodologies were too complex for widespread application. In the late 1980s and early 1990s the development of transvaginal ultrasound guided follicular aspiration (Pieterse et al., 1988, 1991), known as ovum pick-up (OPU), provided for the possibility of a steady source of oocytes from genetically superior females. Furthermore, the abundant supply of abattoir derived oocytes fueled intensive research leading to a deeper understanding of gamete and embryo biology and the consequent development of *in vitro* maturation (IVM), IVF, and *in vitro* embryo culture (IVC) media and conditions necessary for IVEP (Hansen, 2006; Lonergan and Fair, 2016; Sirard, 2018; Ferré et al., 2020; Krisher and Herrick, 2024). Altogether, these advancements established the foundation for current IVEP technologies and the possibility for its large-scale commercial utilization.

Undoubtedly, the use of IVEP in cattle offers several advantages over other assisted reproductive technologies, such as multiple ovulation and embryo transfer (MOET). These include the ability to produce embryos from prepubertal and pregnant females, the potential for greater embryo production per unit of time, more efficient use of semen, and greater ease in incorporating sex-sorted semen, among others (Pontes et al., 2009; Baruselli et al., 2016; Ferré et al., 2020). Nevertheless, the introduction of genomic-assisted selection has been arguably the main driver for the remarkable increase in IVEP utilization. Genomic-assisted selection has allowed for marked improvements in the rate of genetic gain by providing greater selection accuracy (García-Ruiz et al., 2016; Wiggans and Carrillo, 2022). Embryo production technologies, like IVEP, act synergistically with genomic-assisted selection in accelerating genetic progress by enabling greater selection intensity, and reduced generation intervals (Schefers and Weigel, 2012; Mueller and van Eenennaam, 2022). Accordingly, the production and transfer of bovine embryos has increased ~2-fold since the release of the first genomic evaluation in 2009 (Viana, 2024). Moreover, this increase was accompanied by a marked shift towards the utilization of IVEP, with the number of embryos produced and transferred using this technique increasing ~5-fold and ~4-fold, respectively, between 2009 and 2023. Currently, a total of 2.2 million embryos are produced worldwide of which 83.5% are obtained through IVEP (Viana, 2024).

**Figure 1:**
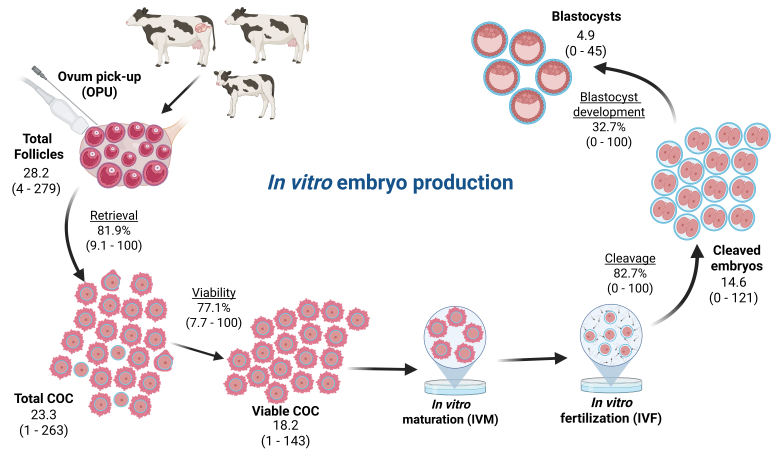
Overview of the in vitro embryo production process and mean (range) outcomes from 3,389 Holstein female donors of various ages and reproductive status (prepubertal, peripubertal, pubertal, and pregnant). Created in BioRender by García-Guerra (2025).

The sustained growth of IVEP in cattle clearly indicates the inherent value of this assisted reproductive technology. Nevertheless, IVEP is not without significant challenges and continues to be a largely inefficient and costly process (Hansen, 2023). Figure 1 depicts an overview of the OPU/IVEP process and provides mean outcomes for each of the steps involved, based on the retrospective analysis of data from 3,389 Holstein donors in a commercial ET facility. It is readily apparent that the overall efficiency of the process is low considering that on average ~18% (range 0 to 100%) of the follicles aspirated will yield a transferable embryo. The success and efficiency of IVEP largely depends on the quantity of oocytes retrieved during OPU as well as the developmental competence (i.e., quality) of these oocytes (Baruselli et al., 2016). As expected, there is marked variability in the quantity and quality of cumulus-oocyte-complexes (COC) among female donors which in turn leads to large variation in the number of viable embryos produced per OPU procedure (Demetrio et al., 2020; Motta et al., 2025a). While this variability is detrimental to the predictability of ET programs, it also highlights the existence of donors that produce a large number of fully competent oocytes capable of yielding viable embryos with near perfect efficiency. This in turn emphasizes the gaps in our understanding of the factors that drive embryo output and more importantly the opportunity for further optimization of IVEP systems. Given the donor’s pivotal role as the source of oocytes, this review aims to summarize and discuss current strategies in donor selection and management that contribute to improving the efficiency of OPU/IVEP programs in cattle.

## Manipulation of ovarian function to enhance oocyte quantity and quality

The complex interactions between the oocyte and follicular somatic cells (Knight and Glister, 2006; Scaramuzzi et al., 2011; Hsueh et al., 2015) underscore the importance of follicular development on oocyte quantity and quality. Accordingly, extensive research has been conducted to understand the interplay between follicular development and both oocyte yield and developmental competence (reviewed by Motta et al., 2025a). Oocyte developmental competence is acquired when follicles reach 2 to 3 mm in diameter, and is positively associated with follicular size (Pavlok et al., 1992; Lonergan et al., 1994; Blondin et al., 1997a; Hagemann et al., 1999; Lequarre et al., 2005; Caixeta et al., 2009; Sarwar et al., 2020a). The greater blastocyst development rates obtained with oocytes retrieved from dominant sized follicles (Hagemann et al., 1999; Caixeta et al., 2009) suggests that improved developmental competence may be associated with the acquisition of dominance. In addition, follicular dominance is temporally associated with the transition from an FSH to an LH dominated milieu which drives follicular differentiation and increased chromatin condensation which are necessary for attainment of developmental competence (Landry and Sirard, 2018; Luciano and Sirard, 2018). As a result, promoting the presence of follicles with a dominant-like phenotype, through reproductive management interventions, can contribute to enhance oocyte quality and thus embryo yield.

### Optimization of follicular development through ovarian superstimulation

Under physiological conditions follicle selection restricts the number of follicles that can acquire a dominant-like phenotype (García-Guerra et al., 2018c). The demonstrated capacity of exogenous FSH to rescue subordinate follicles from atresia (García-Guerra et al., 2015), supports the use of superstimulation to optimize follicular development and enhance oocyte developmental competence. As a result, a large number of studies have been conducted to evaluate the effect of FSH administration on OPU/IVEP outcomes (Table 1). Although there is variability in the reported effects, results from most studies indicate a marked advantage of ovarian superstimulation in embryo yield in agreement with a recent meta-analysis (Sarwar et al., 2020b). Furthermore, our research group recently performed a retrospective analysis of data collected from cyclic and pregnant Holstein heifers (n = 1,037) at a commercial ET facility to evaluate the effect of superstimulation on IVEP (Table 2). Administration of FSH was associated with a significant increase in the total number of follicles aspirated (+11%), number of COC retrieved (+12%), percentage of viable COC (+18%), blastocyst development rates (+24%), and number of blastocysts (+64%). Collectively, these results support the use of superstimulation as a management intervention to improve the overall efficacy of IVEP and, explain the widespread adoption of this practice among practitioners in Europe and North America (Viana, 2024).

**Table 1:** Effect of FSH administration before ovum pick-up and *in vitro* embryo production on number of cumulus oocyte complexes (COC), embryo rate, and number of embryos (mean ± SEM).

**Breed**	**Category**	**COC (n)**			**Embryo (%)**			**Embryo (n)**		**Study**
		**No FSH**	**FSH**		**No FSH**	**FSH**		**No FSH**	**FSH**	
*Bos Taurus*										
Beef	Cow	4.6 ± 1.9^b^	10.6 ± 4.5^a^		-	-		0.6 ± 0.8^b^	2.1 ± 1.2^a^	Chaubal et al. (2006)
		6.4 ± 0.7	8.5 ± 0.6		-	-		1.2 ± 0.3	1.8 ± 0.3	Ongaratto et al. (2020)
		15.6 ± 2.5^a^	12.7 ± 1.4^b^		18.3%^b^	33.1%^a^		3.2 ± 0.8	3.9 ± 0.7	Sola et al. (2023a)
	Cow/Heifer	4.1 ± 3.1^b^	11.8 ± 8.2^a^		17.0%^b^	29.0%^a^		0.7 ± 1.2^b^	3.4 ± 3.9^a^	De Roover et al. (2008)
	Heifer	5.6 ± 1.2	6.1 ± 1.2		22.0%^b^	39.0%^a^		1.0 ± 0.3^b^	2.1 ± 0.4^a^	Goodhand et al. (1999)
Dairy	Cow	19.5 ± 1.6	18.5 ± 1.3		23.5%	26.4%		4.1 ± 0.5	4.3 ± 0.5	Silva et al. (2017)
		26.0 ± 12.7	19.0 ± 9.4		24.2%^b^	68.1%^a^		4.3 ± 2.9^b^	12.8 ± 8.7^a^	Imai et al. (2007)
		20.5 ± 2.2	16.0 ± 2.2		12.2%	17.1%		2.6 ± 0.5	3.0 ± 0.5	Oliveira et al. (2016)
		13.1 ± 1.0^b^	16.5 ± 1.2^a^		25.9%	30.3%		2.4 ± 0.5	3.7 ± 0.7	Vieira et al. (2016)
		12.0 ± 1.6	10.3 ± 1.5		19.8%^b^	34.5%^a^		1.8 ± 0.4^b^	3.0 ± 0.5^a^	Vieira et al. (2014)
	Heiferγ	10.9 ± 0.8	13.5 ± 1.0		26.1% ^b^	35.5%^a^		2.4 ± 0.3^b^	4.0 ± 0.5^a^	Hayden et al. (2023)
		10.9 ± 0.8^b^	16.4 ± 1.1^a^		26.1% ^b^	39.2%^a^		2.4 ± 0.3^b^	5.2 ± 0.6^a^	Hayden et al. (2023)
DP	Heifer	13.2	14.8		42.9%	40.3%		4.1 ± 0.9	4.8 ± 1.1	Scarlet et al. (2023)
*Bos indicus*										
Dairy	Cow	-	-		-	-		2.8^b^	5.8^a^	Elliff et al. (2019)
		11.7 ± 1.9	12.0 ± 2.2		-	-		2.3 ± 0.7^b^	4.1 ± 0.8^a^	Cedeño et al. (2023)
		18.3 ± 1.7	15.0 ± 1.1		-	-		2.9 ± 0.4^b^	3.9 ± 0.2^a^	Ocampo et al. (2024)

γ Indicates pregnant heifers. ^a,b^Different superscripts within a row and outcome indicate differences between treatments (P ≤ 0.05). Adapted from Motta et al. (2025a).

**Table 2:** Ovarian response, cumulus oocyte complex (COC) retrieval, and *in vitro* embryo production of cyclic and pregnant Holstein heifers (13-24 months) with or without FSH stimulation before OPU and IVEP. Ovarian superstimulation was initiated at follicular wave emergence and consisted of 280 or 350 IU of porcine FSH (Folltropin, Vetoquinol USA) distributed in 4 or 5 administrations. Ovum pick-up was performed ~ 40 h after the last FSH administration. Data are presented as LSM ± SEM.

	**Non-stimulated**	**Superstimulated**	**P-value**
N	350	687	
Total follicles	24.5 ± 1.6	27.3 ± 1.7	0.002
*Follicle size distribution (%)*			
Small Follicles (< 6 mm)	77.6 ± 1.2	30.7 ± 0.8	< 0.0001
Medium Follicles (6-10 mm)	14.9 ± 1.0	50.1 ± 0.8	< 0.0001
Large Follicles (> 10 mm)	7.6 ± 0.5	19.1 ± 0.6	< 0.0001
*COC retrieval*			
Total COCs (n)	20.6 ± 1.4	23.1 ± 1.5	0.01
COC retrieval (%)^1^	84.8 ± 1.2	87.0 ± 0.9	0.01
Viable COC (n)2	14.6 ± 1.0	19.0 ± 1.1	< 0.0001
Viable COC (%)3	71.3 ± 2.0	84.0 ± 1.2	< 0.0001
*In vitro embryo production*			
Cleavage (%)4	86.8 ± 1.2	88.1 ± 0.8	0.24
Blastocyst (%)5	38.4 ± 1.8	47.7 ± 1.3	< 0.0001
Blastocysts (n)	4.4 ± 0.3	7.2 ± 0.3	< 0.0001

1 Number of total COC retrieved/number of total follicles; ^2^ Number of COC with a homogenous cytoplasm and at least one layer of compacted cumulus cells; ^3^Number of viable COC/number of total COC; ^4^Number of cleaved embryos/number of viable COC; ^5^Number of transferable blastocysts/number of cleaved embryos.

The ability of ovarian superstimulation to enhance embryo yield following IVEP is attributed to a greater number of COC retrieved and/or enhanced oocyte quality. Intriguingly, results from multiple studies indicate that FSH administration leads to a greater total number of follicles at OPU (De Roover et al., 2008; Vieira et al., 2016; Ongaratto et al., 2020; Hayden et al., 2023). The increase in total number of follicles aspirated may be due to rescue of very small (i.e., 1 - 2 mm) antral follicles (Jaiswal et al., 2004; García-Guerra et al., 2015), and easier follicle visualization due to greater follicle size (Pieterse et al., 1988). Despite initial concerns that the greater follicle size after FSH stimulation could reduce COC retrieval efficacy (Pieterse et al., 1991; Seneda et al., 2001), results from multiple studies indicate that superstimulation does not compromise COC retrieval (Vieira et al., 2016; Silva et al., 2017; Hayden et al., 2023; Scarlet et al., 2023). It is reasonable to speculate that use of superstimulation before OPU has led to adjustments in the retrieval procedure and greater technical proficiency that could overcome the reduced retrieval efficacy associated with larger follicle size. Therefore, the greater number of COCs retrieved following superstimulation with FSH (De Roover et al., 2008; Vieira et al., 2016; Hayden et al., 2023) is the result of increased number of follicles available for aspiration.

The positive effects of superstimulation on oocyte quality include an increase in the percentage of viable or high-quality COC and improved embryo development rates. For example, the percentage of high-quality COC was ~2-fold greater for superstimulated (35.8% ± 2.7) than non-stimulated (17.4% ± 1.8) Holstein heifers, according to the retrospective analysis presented in Table 2 and in agreement with the results of a recent meta-analysis (Sarwar et al., 2020b). As expected, high-quality COC have better developmental competence (Demetrio et al., 2022). Consequently, improved cleavage and/or blastocyst rates after ovarian superstimulation have been reported in many studies (Goodhand et al., 1999; Imai et al., 2007; Vieira et al., 2014, 2016; Hayden et al., 2023) providing further support to the notion that FSH enhances developmental competence. The greater oocyte quality following superstimulation with FSH appears to be strongly associated with an optimization of follicular development since superstimulation increases the proportion of medium (6-10 mm) sized follicles which is positively correlated with oocyte developmental competence (Nivet et al., 2012). The precise cellular and molecular mechanisms by which FSH administration improves developmental competence remain incompletely defined; however, these appear to include: 1) greater ability of cumulus cells to expand during IVM (Sugimura et al., 2017); 2) more favorable chromatin configuration (Soares et al., 2020a, b); and 3) a transcriptomic profile in cumulus cells that favors cell-to-cell communication and an anti-inflammatory response (Sugimura et al., 2017), among others.

The premise underlying the capacity of FSH stimulation to augment the overall efficacy of IVEP is the optimization of follicular development. This is achieved by enabling multiple follicles to acquire a dominant-like phenotype that mirrors the physiological changes occurring as a follicle approaches ovulation, ultimately leading to the acquisition of developmental competence (Landry and Sirard, 2018; Sirard, 2022). However, the implementation of ovarian superstimulation necessitates refinement and optimization to maximize its efficiency. Superstimulation protocols used prior to OPU/IVEP typically involve lower doses and shorter durations of FSH compared to those used for MOET. Additionally, attainment of ovulatory capacity is not required, and oocyte maturation is performed *in vitro*. Modifications to ovarian superstimulation protocols have been extensively investigated. A summary of the main findings is presented in the following sections, with emphasis on the impact of these adjustments on oocyte yield and quality, aiming to provide practical recommendations for the effective application of FSH stimulation by ET practitioners.

#### FSH dosage

Experience gained through the use of superstimulation for MOET indicates that FSH increases superovulatory response and embryo yield in a dose-dependent, albeit limited, manner (Pawlyshyn et al., 1986; Gonzalez et al., 1990; Souza et al., 2007). Considering the differences between MOET and OPU/IVEP we recently conducted two studies to evaluate the effect of FSH dosage on IVEP outcomes in pregnant Holstein heifers (Hayden et al., 2021, 2023). In a first study, administration of 525 IU of FSH resulted in significantly greater number of follicles (24.3 ± 0.9 vs 22.3 ± 0.7), total COCs (14.7 ± 1.0 vs 12.8 ± 0.7), cleavage rate (71.5% ± 3.7 vs 63.8% ± 4.3) and number of blastocysts (3.6 ± 0.7 vs 2.7 ± 0.4) compared to administration of 280 IU (Hayden et al., 2021). Subsequently, we conducted a large dose response study that included three FSH dosages (0, 280 and 525 IU) distributed in 4 administrations (Hayden et al., 2023). Although there were quadratic effects of FSH dose on the number and proportion of medium (6-10 mm) and large (> 10 mm) sized follicles, suggesting a dose-limited superstimulatory response, the number of total follicles increased in a linear dose-dependent manner. Consequently, the number of COC increased linearly with the increase in FSH dosage. More importantly, both cleavage and blastocyst rate increased in a linear dose dependent manner, suggestive of greater developmental competence with increased FSH. The greater COC yield and enhanced developmental competence led to a linear dose dependent increase in embryo yield, whereby administration of 525 IU increased blastocyst yield by 2.2- and 1.3-fold compared to administration of 0 and 280 IU, respectively. Interestingly, FSH dosage was without effect on embryo quality. These results provide strong evidence for an FSH dose dependent increase in IVEP efficacy, albeit the effect of dosages in excess of 525 IU remains to be determined.

#### FSH dose schedule

Administration of FSH is often performed using a decreasing dose schedule to mimic the decrease in circulating FSH concentrations that occurs during the follicular wave. Although use of a decreasing dose schedule can improve superovulatory response during MOET, results from most studies indicate comparable embryo development rates between constant and decreasing dose schedules (Mapletoft and Murphy, 1988; Folchini et al., 2021; Sola et al., 2023b). In the context of OPU/IVEP, we recently conducted a study to evaluate the effect of using a decreasing, constant, or increasing dose schedule on IVEP outcomes in pregnant heifers receiving 350 IU of porcine FSH (Motta et al., 2022). Total number of follicles was not different between the decreasing (28.8 ± 1.8), constant (27.5 ± 2.1), and increasing (29.3 ± 2.1) groups. Even though use of an increasing dose schedule (19.6 ± 1.8) tended to yield more COCs compared to the decreasing (17.0 ± 1.7) and constant (17.1 ± 1.3) dose schedules, there were no differences in the number or percentage of viable COCs. Furthermore, comparable blastocyst rates were observed among the decreasing (41.2% ± 5.3) constant (37.2% ± 4.2) and increasing (40.2% ± 3.5) dose schedules, leading to similar embryo yield. Therefore, maintaining a constant dose schedule may be advisable to reduce the potential for errors.

#### Time of FSH administration

The greater superovulatory response and embryo production observed when initiating FSH treatments at follicular wave emergence during MOET (Nasser et al., 1993; Adams et al., 1994), highlights the need for synchronizing follicular wave emergence before superstimulation for MOET and by extension for OPU/IVEP. Brogliatti et al. (1997) reported a larger number of follicles and COCs at OPU when superstimulation was initiated at follicular wave emergence rather than in the presence of a dominant follicle in prepubertal beef heifers. However, the effect of the time of FSH administration on IVEP was not reported. Consequently, our research group recently evaluated the effect of initiating FSH administration at the time of follicular wave emergence as opposed to random stages of the follicular wave on IVEP outcomes (Hayden et al., 2022). While the number of follicles aspirated and COCs retrieved was not different, the initiation of FSH treatments at follicle wave emergence resulted in a greater (+23%) percentage of medium sized follicles (6-10 mm) indicating improved superstimulatory response. Moreover, blastocyst rate was markedly greater (48.2% vs 33.4%) when FSH treatments were initiated at follicular wave emergence, which in turn led to a 62% increase in blastocyst yield (8.9 ± 1.0 vs. 5.5 ± 0.9). The enhanced developmental competence may be attributed, at least in part, to a greater proportion of oocytes with a more desirable chromatin configuration (Soares et al., 2020a). Interestingly, synchronization of follicular wave emergence before ovarian superstimulation improved the embryo developmental kinetics resulting in a greater proportion of high-quality blastocysts (Hayden et al., 2022). Therefore, synchronization of follicular wave emergence and initiation of superstimulation at the onset of the follicular wave appears as an essential step to optimize IVEP efficacy. It is important to note, however, that the time of follicular wave emergence is affected by the method utilized for synchronization (Hayden et al., 2025); thus, superstimulation must be initiated accordingly.

#### Duration of FSH administration

Ovarian superstimulation requires multiple administrations of FSH to maintain the required concentrations to sustain follicle growth due to the short half-life of porcine FSH (Donaldson, 1984; Demoustier et al., 1988; Adams et al., 1993; Bó et al., 1994). While ovarian superstimulation regimens for MOET typically involve treatment durations of 4 to 5 days, those utilized for OPU/IVEP are usually 2 to 3 days in duration. Two studies have been conducted to date to evaluate the effect of using a 2- or 3-day FSH regimen on ovarian response and IVEP in non-lactating Holstein cows (Silva et al., 2017) and heifers (Motta et al., 2021). The duration of FSH administration did not affect total number of follicles aspirated, COC yield, oocyte developmental competence, nor embryo production indicating that 2- or 3-day FSH regimens result in similar IVEP efficacy.

The inconvenience of the multiple administration regimen and the need to minimize animal handling has prompted the evaluation of simplified superstimulation protocols. The use of a single intramuscular administration of 105 IU of porcine FSH diluted in saline was found to increase blastocyst development rates (+42 to 73%) and overall blastocyst yield (+34 to 78%) when compared to unstimulated controls in two studies using Gyr cows (Cedeño et al., 2023; Ocampo et al., 2024). This approach, however, appears to be less efficacious than the traditional multiple administration regimen as recently reported by our research group (Sala et al., 2024). Pregnant Holstein heifers were superstimulated with 350 IU of porcine FSH diluted in saline using either a 2-day multiple administration regimen or a single administration. Although there were no differences in the total number of follicles aspirated, the 2-day regimen resulted in a greater superstimulatory response (i.e., greater percentage of medium to large sized follicles) and improved COC retrieval efficacy, leading to a greater number of COC. Furthermore, the 2-day regimen resulted in a greater percentage of viable COC (88.6% ± 1.1 vs. 75.0% ± 1.8) and embryo development rates (36.1% ± 3.5 vs. 25.3% ± 3.0), which led to 1.8-fold increase in embryo production (4.7 ± 0.6 vs. 2.6 ± 0.3) compared to the single regimen (Sala et al., 2024). Thus, a multiple administration regimen is the preferable approach to maximize IVEP efficacy, however, a single administration of FSH in saline may be a suitable alternative to non-stimulated approaches when the use of a multiple administration regimen is not feasible.

The combination of FSH with biodegradable polymers, such as sodium hyaluronate (i.e., hyaluronan), to extend FSH half-life provides yet another approach to simplifying FSH regimens for ovarian superstimulation (Bó and Mapletoft, 2020). The efficacy of a single administration of FSH diluted in 0.5% hyaluronan for OPU/IVEP has been investigated extensively in both beef and dairy *Bos taurus* cattle (Vieira et al., 2016; Ongaratto et al., 2020; Demetrio et al., 2021; Santos et al., 2021; Sola et al., 2023a; Sala et al., 2024). Results from multiple studies indicate that a single administration of FSH in 0.5% hyaluronan results in greater oocyte developmental competence and blastocyst yield when compared to a non-stimulated approach (Vieira et al., 2016; Ongaratto et al., 2020; Santos et al., 2021; Sola et al., 2023a). Furthermore, comparable ovarian response, number of COC retrieved, oocyte developmental competence, and embryo yield have been reported when comparing the use of a single FSH administration in 0.5% hyaluronan with the traditional multiple administration regimen (Vieira et al., 2016; Ongaratto et al., 2020; Demetrio et al., 2021). Conversely, we recently reported greater number of viable COC and embryo production in pregnant heifers superstimulated using a 2-day multiple administration regimen (Sala et al., 2024). These results taken together suggest that the use of FSH in 0.5% hyaluronan provides for an effective and simple superstimulation approach during OPU/IVEP, however, the lesser embryo yield of this strategy in pregnant heifers warrants further research.

#### FSH withdrawal or “coasting”

Initial attempts to improve oocyte developmental competence through ovarian superstimulation prior to IVEP were unsuccessful (Lonergan et al., 1994; Blondin et al., 1996), leading to the hypothesis that FSH stimulation may result in asynchronous development of follicles and oocytes (Sirard, 2011). The acquisition of follicular dominance is characterized by a shift in gonadotropic regulation, wherein the dominant follicle becomes dependent on LH in the context of nadir FSH concentrations (García-Guerra et al., 2018a, b; Gomez-León et al., 2020). Consequently, a FSH withdrawal period after ovarian superstimulation and before OPU, known as “coasting”, was incorporated with the aim of promoting follicular differentiation and oocyte competence (Blondin et al., 1997b; Sirard et al., 1999; Blondin et al., 2002).

Results from early studies suggested improved developmental competence and embryo yield following a 48 h coasting duration compared to shorter (24 to 36 h) or longer (60 to 72 h) durations (Blondin et al., 1997b; Sirard et al., 1999; Blondin et al., 2002), although the optimal coasting appeared to be affected by the superstimulation regimen utilized (Sirard et al., 1999). More recently, Nivet et al. (2012) reported enhanced blastocyst development in lactating dairy cows when utilizing coasting durations of 44 to 68 h after a 3-day superstimulation regimen. Similarly, greater blastocyst rates were observed when implementing a coasting duration of 48 h as opposed to 72 h in Angus cows superstimulated with a single administration of FSH in 0.5% hyaluronan (Sola et al., 2023a). Conversely, Landry et al. (2016a) reported comparable oocyte developmental competence among coasting durations of 19, 30 and 43 h in superstimulated Holstein heifers. While a coasting duration of ~40 to 70 h is commonplace in the industry, the lack of consistent findings is intriguing and warrants further research. Interestingly, different coasting durations are intrinsically linked to differences in follicular lifespan (i.e., interval from follicular wave emergence to OPU), and thus, distinguishing the effect of FSH withdrawal from those potentially derived from differences in follicular lifespan is challenging. Surprisingly, to the best of our knowledge the effect of FSH withdrawal in the context of similar follicular age has not been explored, highlighting the opportunity to deepen our understanding of the follicular contributions to oocyte developmental competence.

## Donor selection

The selection of donors for embryo production, whether MOET or IVEP, has long been recognized as an important contributor of success in ET programs. Many donor related factors can affect oocyte quantity and/or quality and thus the efficacy of IVEP programs. These factors include breed (Baruselli et al., 2021), age (Baruselli et al., 2016; Batista et al., 2016), reproductive and lactational status (Vieira et al., 2014; Baruselli et al., 2016), reproductive history (Sood et al., 2017), nutrition (Sales et al., 2015; Dantas et al., 2019), disease (Dickson et al., 2020), and size of the ovarian reserve (Guerreiro et al., 2014). Although consideration of these factors is undoubtedly important, it is essential to recognize that the selection of donors for IVEP is often based on criteria other than embryo production potential. Nevertheless, awareness of the limitations and opportunities brought about by some of these characteristics can be utilized to more effectively select and manage IVEP donor females.

### Age

The link between age and reproductive development makes consideration of donor age in the context of IVEP important. Furthermore, the utilization of young donors for IVEP has become more prevalent since the introduction of genomic assisted selection, given that this strategy allows for a marked reduction of the generation interval (Granleese et al., 2015). Accordingly, we recently evaluated the effect of age group on IVEP outcomes using data from 2,073 superstimulated Holstein heifers in a commercial ET program (Table 3). Heifers were classified based on age into prepubertal (4 to 7 months), peripubertal (8 to 10 months) and pubertal (11 to 17 months). The number of follicles at OPU decreased with age, albeit the greatest reduction was observed between prepubertal and peripubertal heifers consistent with previous reports (Landry et al., 2016b; Viana et al., 2024). As expected, the greater number of follicles in prepubertal heifers led to greater COC yield despite a slight reduction in COC retrieval efficacy (Table 3). Interestingly, age was positively associated with enhanced superstimulatory response as indicated by the greater percentage of medium (6-10 mm) and large (> 10 mm) sized follicles. Differences in the superstimulatory response between age groups are likely associated with the maturation of the hypothalamus-pituitary-gonadal axis that result in increased LH pulsatility allowing for continuous growth of the dominant follicle (Kinder et al., 1995; Adams, 1999). The percentage of viable COC, as well as cleavage and embryo developments rates, were lower in younger heifers (Table 3), consistent with the well-established reduced developmental competence of oocytes from prepubertal heifers (Revel et al., 1995; Baruselli et al., 2016; Landry et al., 2016a; Viana et al., 2024). Consequently, embryo yield was markedly greater for pubertal than both prepubertal and peripubertal heifers (+107% and +45%, respectively) in agreement with previous reports (Viana et al., 2024). Conversely, Landry et al. (2016a) reported similar embryo yield among superstimulated heifers of 5 to 18 months of age. Although embryo development rates were less for heifers of 5 to 10 months of age, the greater number of follicles and COC retrieved compensated for the lower efficacy.

**Table 3:** Ovarian response, cumulus oocyte complex (COC) retrieval, and *in vitro* embryo production of superstimulated non-pregnant Holstein heifers of different ages. Ovarian superstimulation was performed using 280 or 350 IU of porcine FSH (Folltropin, Vetoquinol USA). Data are presented as LSM ± SEM.

	**Heifers**			**P-value**
	**Prepubertal** **(4 to 7 mo)**	**Peripubertal** **(8 to 10 mo)**	**Pubertal** **(11 to 17 mo)**	
N	582	934	557	
*Ovarian response*				
Total follicles	37.1 ± 0.9^a^	28.2 ± 0.6^b^	26.5 ± 0.7^c^	< 0.0001
Small Follicles (< 6 mm; %)	46.2 ± 1.5^a^	37.8 ± 1.4^b^	30.8 ± 1.3^c^	< 0.0001
Medium Follicles (6-10 mm; %)	44.3 ± 1.3^c^	50.0 ± 1.3^b^	54.1 ± 1.4^a^	< 0.0001
Large Follicles (> 10 mm; %)	6.2 ± 0.3^c^	8.5 ± 0.4^b^	10.7 ± 0.5^a^	< 0.0001
*COC retrieval*				
Total COCs (n)	29.9 ± 0.8^a^	22.7 ± 0.5^b^	22.3 ± 0.6^b^	< 0.0001
COC retrieval (%)1	83.3 ± 0.7^b^	83.1 ± 0.6^b^	85.9 ± 0.6^a^	< 0.0001
Viable COC (n)2	22.7 ± 0.8^a^	17.8 ± 0.5^b^	18.7 ± 0.6^b^	< 0.0001
Viable COC (%)3	77.9 ± 0.8^c^	80.5 ± 0.7^b^	85.2 ± 0.6^a^	< 0.0001
*In vitro embryo production*				
Cleavage (%)4	71.1 ± 1.1^c^	80.5 ± 0.8^b^	83.9 ± 0.7^a^	< 0.0001
Embryo (%)5	17.6 ± 0.7^c^	28.5 ± 0.9^b^	38.2 ± 1.0^a^	< 0.0001
Embryo (n)	2.8 ± 0.2^c^	4.0 ± 0.2^b^	5.8 ± 0.3^a^	< 0.0001

Different superscript letters within a row indicate differences between age groups ^a,b^ (P ≤ 0.05). ^1^Number of total COC retrieved/number of total follicles; ^2^Number of COC with a homogenous cytoplasm and at least one layer of compacted cumulus cells; ^3^Number of viable COC/number of total COC; ^4^Number of cleaved embryos/number of viable COC; ^5^Number of transferable embryos/number of cleaved embryos.

The use of FSH-based superstimulation for IVEP in young prepubertal heifers is commonplace, particularly among *Bos taurus* donors (Baruselli et al., 2016; Landry et al., 2016a; Currin et al., 2017; Viana et al., 2024). The unique endocrine and follicular differences along with the lesser oocyte developmental competence between prepubertal and postpubertal female cattle make the use of ovarian stimulation a logical approach. Results from multiple studies in prepubertal Holstein heifers indicate positive effects of FSH-stimulation on COC yield and/or oocyte developmental competence (Presicce et al., 1997; Batista et al., 2016; Currin et al., 2017), albeit these do not consistently result in improved embryo yield. Furthermore, the precise superstimulatory protocol utilized appears to be important, for example, utilization of a 3-day superstimulation regimen in 2 to 6 months old heifers improved superstimulatory response and blastocyst development rates when compared to a 1.5-day superstimulation regimen (Currin et al., 2017). The reduced superstimulatory response and IVEP efficiency observed in young prepubertal and peripubertal heifers, even following superstimulation, highlights the need for further optimization of ovarian superstimulation protocols. It is important, however, that these efforts carefully consider the physiological differences of prepubertal heifers at various stages of development, as well as the oocyte retrieval methodology, and IVEP system (Viana et al., 2024).

### Pregnancy status

As previously mentioned, one of the unique advantages of IVEP is the ability of continued embryo production even during pregnancy. Pregnancy is accompanied by distinct endocrine and follicular differences which can affect IVEP efficacy. Although the wave-like pattern of follicle development continues during pregnancy, elevated circulating progesterone concentrations inhibit LH pulsatile secretion leading to reduced dominant follicle growth (Adams et al., 1992; Bergfeld et al., 1996). As a result, follicular waves occur more frequently and maximal dominant follicle size is smaller as gestation advances (Ginther et al., 1989, 1996). The frequent utilization of pregnant donors for OPU/IVEP makes it necessary to better understand the potential impact of the physiology of pregnancy on IVEP outcomes.

We recently evaluated the effect of pregnancy status on IVEP outcomes using data from superstimulated Holstein heifers (12 to 24 months). Although there were no differences in the number of follicles aspirated between pregnant and cyclic non-bred heifers (Table 4), pregnant heifers had a slight but significant increase in the percentage of large (> 10 mm) sized follicles suggestive of greater superstimulatory response. Pregnancy status was without effect on COC retrieval efficacy, which, coupled with the lack of differences in the total number of follicles, resulted in a comparable number of COC between pregnant and cyclic heifers. Interestingly, Baruselli et al. (2016) reported reduced COC yield in non-stimulated pregnant than pubertal heifers, although this could be confounded by age, since pubertal heifers (10-12 months) were markedly younger than pregnant heifers (14 to 18 months). More importantly, pregnant heifers had greater (+20%) embryo development rates, indicating enhanced oocyte developmental competence, which led to a corresponding increase in number of transferable embryos and thus greater overall IVEP efficacy in agreement with a previous report in non-stimulated Holstein heifers (Baruselli et al., 2016).

**Table 4:** Ovarian response, cumulus oocyte complex (COC) retrieval, and *in vitro* embryo production of superstimulated cyclic and pregnant Holstein heifers (12 to 24 months of age). Ovarian superstimulation was performed using 280 or 350 IU of porcine FSH (Folltropin, Vetoquinol USA). Data are presented as LSM ± SEM.

	**Reproductive status**			**P-value**
	**Cyclic**		**Pregnant**	
N		278	590	
Age		14.4 ± 0.2	17.1 ± 0.1	< 0.0001
*Ovarian response*				
Total follicles		25.5 ± 0.7	26.0 ± 0.6	0.54
Small Follicles (< 6 mm; %)		34.2 ± 1.5	27.7 ± 1.2	< 0.0001
Medium Follicles (6-10 mm; %)		51.5 ± 1.6	50.8 ± 1.5	0.48
Large Follicles (> 10 mm; %)		9.7 ± 0.6	16.0 ± 0.8	< 0.0001
*COC retrieval*				
Total COCs (n)		21.4 ± 0.7	22.2 ± 0.6	0.36
COC retrieval (%)^1^		87.1 ± 0.8	87.3 ± 0.7	0.72
Viable COC (n)2		17.8 ± 0.7	18.7 ± 0.5	0.24
Viable COC (%)3		85.4 ± 0.8	85.5 ± 0.7	0.87
*In vitro embryo production*				
Cleavage (%)4		86.5 ± 0.9	87.4 ± 0.8	0.17
Embryo (%)5		40.7 ± 1.4	48.7 ± 1.2	< 0.0001
Embryo (n)		6.0 ± 0.3	7.4 ± 0.3	0.01

1 Number of total COC retrieved/number of total follicles; ^2^Number of COC with a homogenous cytoplasm and at least one layer of compacted cumulus cells; ^3^Number of viable COC/number of total COC; ^4^Number of cleaved embryos/number of viable COC; ^5^Number of transferable embryos/number of cleaved embryos.

The increased use of pregnant cattle as donors for IVEP has prompted investigations into both the necessity of ovarian superstimulation and the optimal methods for its implementation. As described previously in this review, FSH-stimulation increases COC yield, oocyte developmental competence, and embryo production of pregnant Holstein heifers in a linear dose-dependent manner (Hayden et al., 2023). Furthermore, results from recent studies by our research group indicate that a multiple administration regimen of FSH is preferable to a single administration in pregnant heifers (Sala et al., 2024), albeit the dose schedule utilized during a multiple administration regimen does not affect embryo yield (Motta et al., 2022). In addition, synchronization of follicular wave emergence, using follicular ablation, and initiation of FSH treatments at wave emergence improved superstimulatory response, and enhanced both blastocyst rate and embryo quality (Hayden et al., 2022). Although labor-intensive, follicular ablation results in a less variable interval to wave emergence in pregnant heifers, offering more precise control of follicle development than other methods (Hayden et al., 2025). Collectively, these results allowed for the development of a superstimulation protocol that maximizes IVEP efficacy in pregnant heifers.

### Antral follicle count and anti-Müllerian hormone phenotype

The collection of ovarian follicles of an individual constitute the ovarian reserve which can be divided into the pre-established reserve (i.e., primordial follicles) and the dynamic reserve (i.e., small antral follicles) (Monniaux et al., 2014). Determination of the antral follicle count (AFC), through ultrasonography, permits for the practical assessment of the dynamic reserve. In cattle, AFC is highly repeatable within an individual, however, varies greatly between individuals (Singh et al., 2004; Burns et al., 2005; Ireland et al., 2007). Anti-Müllerian hormone (AMH), is secreted by granulosa cells of large preantral and small antral growing follicles (Durlinger et al., 2002; Rico et al., 2011; Campbell et al., 2012). As a result, strong positive correlations between AFC and circulating AMH have been reported (Ireland et al., 2008; Rico et al., 2009; Batista et al., 2014; García-Guerra et al., 2017; Cardoso et al., 2018), highlighting the ability of AMH to serve as biomarker for the ovarian reserve. Furthermore, circulating concentrations of AMH, like AFC, vary minimally within an individual allowing a single AMH measurement to be used as a predictor of AFC (Ireland et al., 2008, 2011; Rico et al., 2009). As expected, the greater number of follicles in high compared to low AFC/AMH females leads to a greater number of COCs retrieved and thus, greater embryo yield following IVEP (Ireland et al., 2007; Guerreiro et al., 2014; Silva-Santos et al., 2014; Batista et al., 2016). Not surprisingly, the marked variability observed in both AFC and AMH has significant practical implications, enabling the identification of female donors with superior IVEP capabilities.

The concept that AFC/AMH is informative of the number of small antral follicles responsive to FSH, combined with the demonstrated effectiveness of exogenous FSH to rescue subordinate follicles and promote their development toward dominance, explains why AFC/AMH can predict superstimulatory response (Singh et al., 2004; Ireland et al., 2007; García-Guerra et al., 2015). Consequently, results from multiple studies indicate a positive association between AFC/AMH and both superovulatory response and embryo yield during MOET (Ireland et al., 2007; Rico et al., 2009, 2012; Silva-Santos et al., 2014; Souza et al., 2015; Center et al., 2018). In the context of OPU/IVEP, we recently reported positive associations between circulating AMH concentration and the number of follicles, COCs retrieved, and transferable embryos following IVEP in FSH-stimulated cyclic and pregnant heifers (Motta et al., 2025a). As a result, donors can be categorized into distinct AMH classes (i.e., low, intermediate and high) that are associated with marked differences in IVEP outcomes (Table 5). As anticipated, the greater number of follicles available for aspiration in high AMH females leads to a greater number of COC and thus embryo yield. Interestingly, the percentage of medium-sized (6-10 mm) follicles was greater for high AMH donors than low AMH donors suggesting a differing ability to respond to FSH stimulation. This may be due, at least in part, to differences in gonadotropin responsiveness of follicular cells between cattle with low and high AFC (Mossa et al., 2010; Scheetz et al., 2012).

**Table 5:** Ovarian response, cumulus oocyte complex (COC) retrieval, and *in vitro* embryo production of cyclic (n = 194) and pregnant (n = 255) Holstein heifers with low, intermediate, and high AMH phenotypes. Ovarian superstimulation was performed using 280 or 350 IU of porcine FSH (Folltropin, Vetoquinol USA) distributed in 4 or 6 administrations. Ovum pick-up was performed ~ 40 h after the last FSH administration. Data are presented as LSM ± SEM.

	**AMH phenotype**			**P-value**
	**Low**	**Intermediate**	**High**	
**Cyclic heifers**				
N	64	65	65	
AMH (pg/mL)	120.1 ± 10.5^c^	250.0 ± 10.4^b^	496.9 ± 18.1^a^	< 0.0001
*Ovarian response*				
Total follicles	18.1 ± 0.7^c^	24.3 ± 1.0^b^	34.8 ± 1.2^a^	< 0.0001
Small Follicles (< 6 mm; %)	35.9 ± 2.3^a^	26.9 ± 1.9^b^	24.8 ± 1.8^b^	< 0.0001
Medium Follicles (6-10 mm; %)	48.5 ± 1.9^b^	60.9 ± 1.7^a^	63.0 ± 1.6^a^	< 0.0001
Large Follicles (> 10 mm; %)	14.9 ± 1.5^a^	11.7 ± 1.4^ab^	11.3 ± 1.2^b^	0.02
*COC retrieval*				
Total COCs (n)	10.3 ± 0.6^c^	15.3 ± 0.8^b^	25.1 ± 1.3^a^	< 0.0001
COC retrieval (%)1	57.1 ± 1.8^c^	62.1 ± 1.7^b^	68.5 ± 1.5^a^	< 0.0001
Viable COC (n)2	9.0 ± 0.5^c^	12.4 ± 0.7^b^	20.1 ± 1.0^a^	< 0.0001
Viable COC (%)3	87.8 ± 2.0^a^	82.4 ± 2.4^b^	83.1 ± 2.2^b^	0.01
*In vitro embryo production*				
Cleavage (%)4	69.1 ± 3.2	71.3 ± 2.9	70.0 ± 2.9	0.70
Embryo (%)5	30.5 ± 3.6^A^	36.3 ± 3.6	38.3 ± 3.5^B^	0.07
Embryo (n)	1.8 ± 0.3^c^	3.3 ± 0.5^b^	5.6 ± 0.8^a^	< 0.0001
Grade 1 embryo (%)6	50.9 ± 6.3^b^	74.2 ± 4.4^a^	73.4 ± 4.0^a^	0.0007
**Pregnant heifers**				
N	85	85	85	
AMH (pg/mL)	157.6 ± 10.1^c^	313.4 ± 10.2^b^	570.8 ± 10.0^a^	< 0.0001
*Ovarian response*				
Total follicles	16.6 ± 0.7^c^	23.5 ± 1.0^b^	28.7 ± 1.1^a^	< 0.0001
Small Follicles (< 6 mm; %)	25.9 ± 1.9	23.0 ± 1.6	23.2 ± 1.6	0.18
Medium Follicles (6-10 mm; %)	53.9 ± 1.9^b^	56.3 ± 1.7^ab^	59.8 ± 1.6^a^	0.009
Large Follicles (> 10 mm; %)	18.2 ± 1.5^a^	18.6 ± 1.4^a^	15.3 ± 1.2^b^	0.02
*COC retrieval*				
Total COCs (n)	9.9 ± 0.6^c^	13.4 ± 0.8^b^	18.5 ± 1.0^a^	< 0.0001
COC retrieval (%)^1^	58.9 ± 2.1^b^	56.9 ± 2.0^b^	65.5 ± 1.8^a^	< 0.0001
Viable COC (n)^2^	8.1 ± 0.5^c^	11.4 ± 0.7^b^	16.6 ± 1.0^a^	< 0.0001
Viable COC (%)^3^	82.5 ± 1.7^b^	85.5 ± 1.4^b^	90.3 ± 1.0^a^	< 0.0001
*In vitro embryo production*				
Cleavage (%)^4^	68.3 ± 3.9^b^	79.6 ± 2.9 ^a^	75.4 ± 3.2^a^	< 0.0001
Embryo (%)^5^	61.0 ± 3.8^a^	52.5 ± 3.6^b^	54.8 ± 3.4^b^	0.03
Embryo (n)	3.0 ± 0.4^b,B^	4.0 ± 0.5^b,A^	5.5 ± 0.6^a^	0.0002
Grade 1 embryo (%)^6^	51.3 ± 4.6^b^	70.1 ± 4.3^a^	69.4 ± 3.9^a^	0.0007

Different superscript letters within a row indicate differences between AMH groups ^a,b^ (P ≤ 0.05); ^A,B^(P<0.10). ^1^Number of total COC retrieved/number of total follicles; ^2^Number of COC with homogenous cytoplasm and at least one layer of compacted cumulus cells; ^3^Number of viable COC/number of total COC; ^4^Number of cleaved embryos/number of viable COC; ^5^Number of transferable embryos/number of cleaved embryos; ^6^Number of embryos quality grade 1 based on the IETS guidelines/number of transferable embryos. Adapted from Motta et al. (2025a).

The effect of AFC/AMH phenotype on oocyte developmental competence appears to be poorly understood as indicated by the conflicting observations reported in the literature. Although results from most studies conducted in non-stimulated donors suggest that AFC/AMH is without effect on developmental competence (Ireland et al., 2007; Guerreiro et al., 2014; Silva-Santos et al., 2014), some have suggested that AFC/AMH may be positively associated with developmental competence (Batista et al., 2016; Santos et al., 2016). Furthermore, we recently reported a small but significant positive association between circulating AMH and overall embryo development rate in superstimulated heifers (Motta et al., 2025a). Interestingly, assessment of the effect of AMH class on cleavage and embryo development rates also reveal inconsistent results (Table 5). Even though, cleavage rates did not differ among cyclic heifers with low, intermediate, and high AMH concentrations, embryo development rates tended to be greater for females with high AHM compared to those with low AMH. As a result, overall embryo development rate (transferable embryos/viable COC) increased with increasing AMH (low AMH: 20.2% ± 2.5^b^; intermediate AMH: 25.8% ± 2.6^ab^; high AMH: 26.7% ± 2.6^a^; P = 0.02). Conversely, in pregnant heifers, cleavage rates were greater for intermediate and high AMH heifers, whereas embryo development rates were greater for low AMH heifers. Overall embryo development rates, however, were not different between AMH classes (low AMH: 37.8% ± 3.9; intermediate AMH: 39.5% ± 3.8; high AMH: 36.5% ± 3.6; P = 0.48). The reason for these contrasting observations remains unclear, and thus further research is needed to unravel the potential contributions of AFC/AMH phenotype to oocyte quality. Nevertheless, it is noteworthy that the percentage of high-quality embryos was greater in intermediate and high AMH pregnant and cyclic heifers.

The practicality of measuring AMH offers a valuable opportunity for the early selection of heifers with superior embryo production potential. In a recent study, AMH concentrations measured monthly from birth to one year of age were evaluated for their association with IVEP outcomes at ~365 days in Holstein heifers (Motta et al., 2024). Circulating AMH increased after birth, peaked around 84 days of age, and stabilized after 224 days in agreement with results from previous studies (Mossa et al., 2017). After ~200 days, AMH concentrations were significantly and positively associated with the number of follicles aspirated, number of viable COC, and embryo yield at 365 days of age. In contrast, AMH measured before 196 days showed no or only weak correlations with IVEP outcomes. As a result, classification of heifers based on AMH measured after ~200 days of age revealed consistent and progressive increases in embryo yield at 365 days of age. These findings indicate that AMH can identify yearling donors with superior embryo production potential as early as ~200 days of age coincident with the age at which circulating AMH concentrations become stabilized.

The marked variation in AFC/AMH among individuals coupled with the strong associations between AFC/AMH and embryo production suggest that the large variability in embryo production among donors can be explained by differences in AFC/AMH phenotype. Although donor selection is often based on genetic merit for other production relevant traits, assessment of AFC and/or AMH can complement these criteria to identify donors with superior embryo production potential.

#### Genomic selection for enhanced IVEP

The capacity of IVEP to increase selection intensity has made it an essential tool in progressive breeding programs. Consequently, the ability to estimate genetic merit for embryo production related traits has garnered significant interest, as evidenced by numerous studies (König et al., 2007; Jaton et al., 2016; Vizoná et al., 2020; Zoda et al., 2021; Huang et al., 2023; Machado et al., 2024). Established female traits such as daughter pregnancy rate, cow conception rate, and heifer conception rate (HCR) are commonly used to select for improved reproductive performance, making their potential application in donor selection inherently appealing. Results from a recent study suggest that fertility traits do not affect overall embryo yield (Chasi et al., 2025); however, greater genetic merit for HCR was associated with greater embryo quality. Genetic parameters of embryo production in cattle have been evaluated in Holstein (Jaton et al., 2016; Parker Gaddis et al., 2017; Huang et al., 2023), Gyr (Vizoná et al., 2020; Machado et al., 2024), and Japanese black cattle (Zoda et al., 2021) among other breeds. Collectively results from these studies indicate the presence of genetic variability for embryo production related traits and therefore the potential for selection and improvement. Heritability estimates for embryo production related traits in Holstein cattle vary between studies; however, as expected, they are generally greater for the number of oocytes retrieved than for embryo yield (Merton et al., 2009; Jaton et al., 2016; Parker Gaddis et al., 2017; Huang et al., 2023).

**Figure 2:**
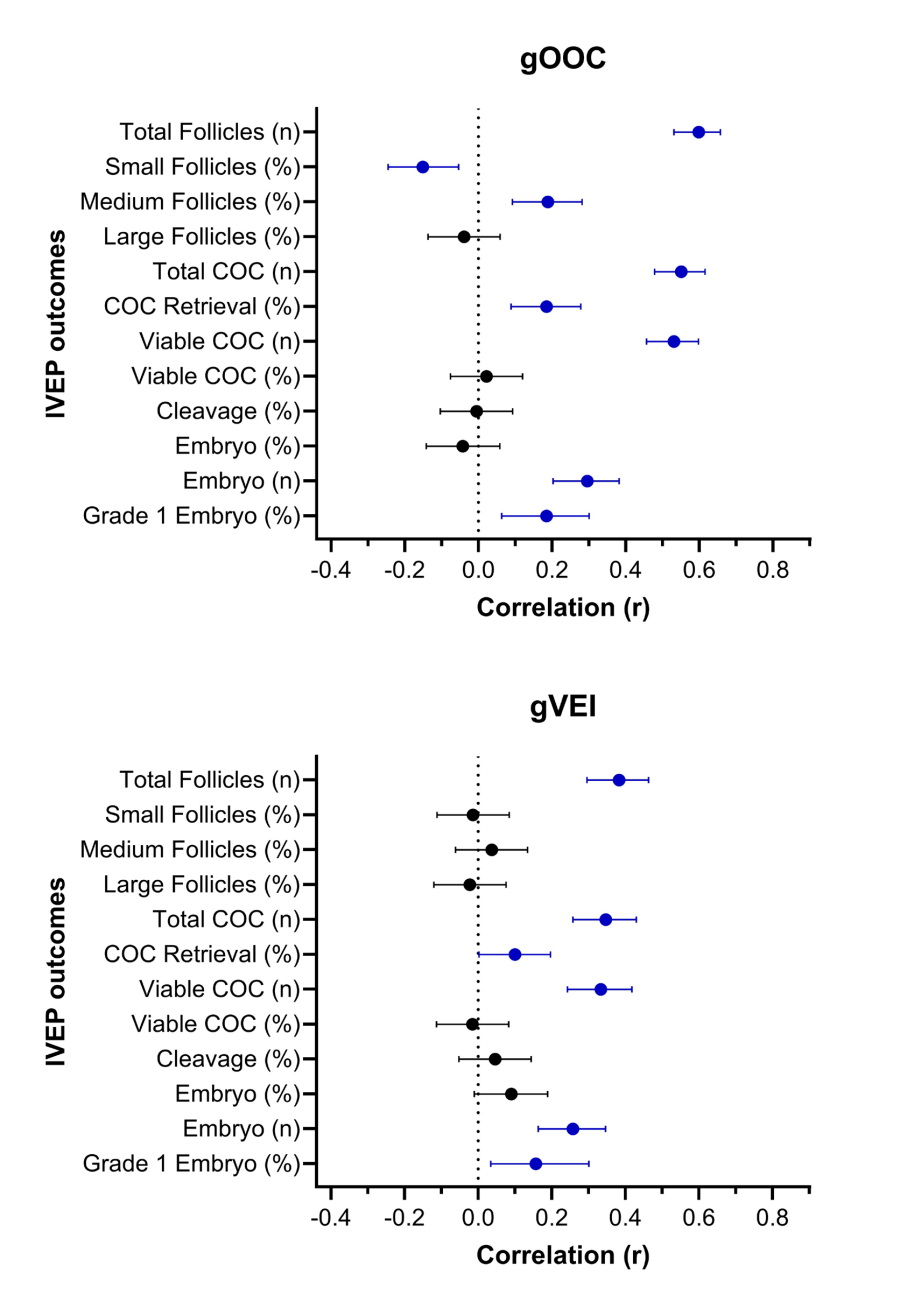
Forest plot of correlation coefficients (95% confidence interval) for the association between genomic merit for total oocytes (gOOC; upper panel) and viable embryos produced in vitro (gVEI; lower panel) with IVEP outcomes in superstimulated cyclic and pregnant heifers (n = 400). Blue symbols indicate P < 0.05.

Advancements in genomic selection, combined with the genetic variability underlying embryo production traits, have led to the commercial availability of traits specifically designed to identify female donors with superior embryo production capabilities. For example, total oocytes (OOC; h^2^ = 0.35) and viable embryos produced *in vitro* (VEI; h^2^ = 0.11) are two recently released, commercially available traits from STgenetics^®^, directed at estimating the genetic potential of Holstein females for COC and embryo yield following IVEP, respectively. Recently, we evaluated the association between the genomic merit for OOC (gOOC) and VEI (gVEI) with ovarian response, COC yield, and embryo production in 400 superstimulated cyclic and pregnant Holstein heifers (Garcia-Guerra and Motta, personal communication). Mean (± SD) for gOOC and gVEI was 15.7 ± 5.1 and 3.9 ± 0.7, respectively. Both gOOC and gVEI were positively correlated with number of total follicles aspirated, number of total COC retrieved, COC retrieval efficacy, number of viable COC, number of transferable embryos, and percentage of grade 1 embryos (Figure 2). As expected, the association between gOOC and number of follicles and COC was stronger than that between gVEI and the same outcomes. Interestingly, gOOC was positively correlated with the percentage of medium (6-10 mm) sized follicles and negatively correlated with the percentage of small (< 6 mm) sized follicles following superstimulation, suggesting improved superstimulatory response. In addition, both gOOC (r = 0.52; P < 0.0001) and gVEI (r = 0.35; P < 0.0001) were positively correlated with circulating concentrations of AMH.

These traits have also been combined along with others into a selection index, termed Donor Output Index (DOI), providing producers and ET practitioners with a tool to select donors with superior IVEP performance. Accordingly, we evaluated circulating AMH concentration, ovarian response, COC yield, and IVEP in superstimulated cyclic and pregnant Holstein heifers with low, intermediate, or high genetic merit for DOI (gDOI). As expected, heifers with greater gDOI had greater circulating AMH concentrations. Furthermore, greater gDOI was associated with marked increases in number of follicles aspirated, total COC, viable COC, and transferable embryos (Table 6). Consequently, the probability of producing more than 2 embryos per OPU session was greater for intermediate (60.5% ± 4.9) and high (74.4% ± 4.3) gDOI heifers compared to low (53.1% ± 5.0) gDOI heifers, supporting the ability of the index to identify donors with greater IVEP capabilities. In addition, greater gDOI was associated with greater superstimulatory response as indicated by the greater percentage of medium (6-10 mm) sized follicles (Table 6). Although there were no major differences in oocyte developmental competence among gDOI classes, the percentage of excellent quality embryos was greater in high gDOI than low gDOI heifers (Table 6). Selection for greater embryo quality has important fertility implications since high-quality embryos yield greater conception rates and have reduced pregnancy loss (Carrenho-Sala et al., 2016; Ferraz et al., 2016). The release of traits and selection indexes specific for IVEP provides a simple and readily available method for donor selection, considering the widespread use of genomic testing in dairy cattle. Furthermore, because these are provided alongside other commonly used production relevant traits and selection indexes, it facilitates their integration into established breeding schemes and ET programs.

**Table 6:** Circulating anti-Müllerian hormones (AMH), ovarian response, cumulus oocyte complex (COC) retrieval, and *in vitro* embryo production of cyclic and pregnant (n = 400) Holstein heifers with low, intermediate, and high genetic merit for Donor Output Index (DOI). Ovarian superstimulation was performed using 280 or 350 IU of porcine FSH (Folltropin, Vetoquinol USA) distributed in 4 or 6 administrations. Ovum pick-up was performed ~ 40 h after the last FSH administration. Data are presented as LSM ± SEM.

	**gDOI**			**P-value**
	**Low**	**Intermediate**	**High**	
N	133	133	134	
gDOI	3.7 ± 0.04^c^	4.9 ± 0.05^b^	6.1 ± 0.04^a^	< 0.0001
AMH (pg/mL)	227.3 ± 24.8^c^	318.6 ± 24.6^b^	459.6 ± 25.6^a^	< 0.0001
*Ovarian response*				
Total follicles	19.5 ± 0.7^c^	24.2 ± 0.9^b^	32.3 ± 1.1^a^	< 0.0001
Small Follicles (< 6 mm; %)	30.6 ± 1.6^a^	23.5 ± 1.3^b^	23.7 ± 1.3^b^	< 0.0001
Medium Follicles (6-10 mm; %)	52.6 ± 1.5^c^	57.4 ± 1.4^b^	62.0 ± 1.3^a^	< 0.0001
Large Follicles (> 10 mm; %)	15.3 ± 1.1^a^	17.4 ± 1.2^a^	12.9 ± 0.9^b^	< 0.0001
*COC retrieval*				
Total COCs (n)	11.5 ± 0.6^c^	15.7 ± 0.8^b^	21.8 ± 1.0^a^	< 0.0001
COC retrieval (%)1	57.6 ± 1.8^c^	64.4 ± 1.7^b^	68.6 ± 1.6^a^	< 0.0001
Viable COC (n)2	9.7 ± 0.5^c^	13.3 ± 0.7^b^	18.3 ± 0.9^a^	< 0.0001
Viable COC (%)3	86.0 ± 1.3	85.5 ± 1.3	85.6 ± 1.2	0.53
*In vitro embryo production*				
Cleavage (%)4	73.1 ± 3.0^ab^	73.6 ± 2.9^a^	69.8 ± 3.1^b^	0.04
Embryo (%)5	48.2 ± 2.9	47.2 ± 2.8	49.0 ± 2.7	0.71
Embryo (n)	3.1 ± 0.3^c^	4.3 ± 0.4^b^	6.0 ± 0.5^a^	< 0.0001
Grade 1 embryo (%)6	53.1 ± 4.0^b^	66.6 ± 3.7^a^	65.9 ± 3.5^a^	0.001

Different superscript letters within a row indicate differences between DOI groups ^a,b^ (P ≤ 0.05). ^1^Number of total COC retrieved/number of total follicles; ^2^Number of COC with homogenous cytoplasm and at least one layer of compacted cumulus cells; ^3^Number of viable COC/number of total COC; ^4^Number of cleaved embryos/number of viable COC; ^5^Number of transferable embryos/number of cleaved embryos; ^6^Number of embryos quality grade 1 based on the IETS guidelines/number of transferable embryos.

## Conclusions and future directions: development of targeted donor management strategies

Although substantial effort has been dedicated to improving IVEP methodologies, these advances have resulted in only modest gains in efficiency, as recently highlighted by Hansen (2023). This challenge underscores the need for continued innovations in the ET industry aimed at maximizing the overall efficacy of IVEP. The growing body of research supporting ovarian superstimulation prior to OPU highlights its pivotal role in improving IVEP efficiency in cattle. Nevertheless, the large variation among donors in terms of oocyte quantity and quality – and consequently embryo yield – remains a significant challenge. This variability is strongly associated with AFC/AMH allowing for identification of female donors with superior embryo production capabilities. Furthermore, the recent availability of embryo production specific traits and selection indexes provides an additional tool for producers and ET practitioners to select superior embryo donors, further enhancing the synergy between genomic assisted selection and IVEP. The continuous search for ways to enhance IVEP efficacy has deepened our understanding of the physiological mechanisms underlying the large variation in embryo yield, while also highlighting the potential to exploit these differences through targeted donor management strategies. For example, utilizing AFC/AMH phenotypes offers a physiologically relevant approach to developing refined ovarian superstimulation regimens aimed at optimizing IVEP efficacy.

Variations in AFC/AMH phenotype in cattle are accompanied by marked physiological differences that include greater circulating concentrations of FSH (Singh et al., 2004; Burns et al., 2005; Ireland et al., 2007) and a diminished capability of follicular cells to respond to gonadotropin stimulation (Mossa et al., 2010; Scheetz et al., 2012) in low AFC/AMH females. For example, granulosa cells from low AFC/AMH females have reduced CYP19A1 mRNA abundance and estradiol secretion following stimulation with FSH (Scheetz et al., 2012). These findings suggest that low and high AFC/AMH donors may respond differently to ovarian superstimulation regimens. Accordingly, recent reports indicate that administration of an excessive dosage of FSH (1680 IU) to low AFC heifers results in follicles with altered endocrine characteristics and diminished ovulatory capacity (Karl et al., 2021; Clark et al., 2022), as well as a large proportion of expanded COCs that perform poorly during IVEP (Karl et al., 2023), suggesting that reduced FSH dosages may be needed for superstimulation of low AFC/AMH females. Accordingly, our research group has focused on evaluating the effect of different aspects of ovarian superstimulation in the context of AFC/AMH phenotype. For example, we recently evaluated the effect of three different FSH doses (0, 280, and 525 IU) on IVEP outcomes in high (Motta et al., 2023) and low (Motta et al., 2025b) AMH pregnant heifers. Administration of FSH before OPU enhanced IVEP in both high- and low-AMH heifers in a dose-dependent manner, with embryo yield increases, relative to the non-stimulated control, of­ ~146% and ~133% for low and high AMH heifers, respectively. Interestingly, however, maximal oocyte developmental competence was attained at a lesser FSH dosage in low than high AMH heifers, suggesting that the optimal total FSH dosage is less for low than high AMH heifers. Extending the duration of FSH administration while maintaining the total dosage results in a lower daily FSH dose, offering a potential approach for the superstimulation of low AFC/AMH females. Consequently, we evaluated the effect of FSH treatment duration on ovarian response and embryo yield during MOET in low and high AMH ewes (Brochado et al., 2024). Remarkably, lengthening the superstimulatory treatment from 3 to 4 days markedly increased embryo production in low but not in high AMH ewes. Furthermore, such increase in embryo yield was primarily attributed to greater fertilization and embryo development rates, suggestive of enhanced oocyte developmental competence. Collectively, these findings underscore the potential for superstimulatory regimen refinement based on AMH phenotype to maximize IVEP efficacy.

The development of targeted donor reproductive management strategies for OPU/IVEP based on AFC/AMH phenotype and/or other characteristics like genetic merit for embryo production related traits is inherently appealing. Leveraging specific donor characteristics to guide the choice of FSH treatment regimen offers practical advantages, enabling the optimization of IVEP outcomes while promoting more efficient use of FSH, which remains a costly input. Although results presented herein appear promising and suggest that ovarian superstimulation regimens can be optimized in the context of AFC/AMH phenotype, the widespread application of this approach requires further research. In addition, it is imperative to evaluate the potential of additional donor-specific traits for use in the systematic refinement of donor management strategies, with the long-term goal of enhancing IVEP efficacy in a manner that is cost-effective and biologically sound.

## Data Availability

Research data is available in the body of the article.
